# Draft genome sequence of stress-tolerant *Rhodococcus pyridinivorans* IEGM 639

**DOI:** 10.1128/mra.00260-26

**Published:** 2026-04-23

**Authors:** Anastasiia Golysheva, Anastasiia Krivoruchko, Maria Kuyukina, Irina Ivshina

**Affiliations:** 1 Perm Federal Research Center, Perm, Russia; 2 Perm State University 64949, Perm, Russia; University of Manitoba, Winnipeg, Manitoba, Canada

**Keywords:** stress-tolerant microorganisms, resistance to solvents, *Rhodococcus pyridinivorans*, biotechnology, bioremediation

## Abstract

A genome of *Rhodococcus pyridinivorans* IEGM 639 was sequenced using the Illumina technology. This bacterium is resistant to solvents, heavy metals, and high salinity and oxidizes a variety of organic compounds. The obtained sequence can be used to reveal genetic mechanisms of these properties.

## ANNOUNCEMENT

Stress-tolerant and undemanding microorganisms are easy to maintain and suited for long-term biotechnological processes and bioremediation *in situ* (1–3). *Rhodococcus pyridinivorans* IEGM 639 was isolated from the oilfield snow, Perm region, Russia. The snow was taken aseptically. After melting, 100 μL was spread on the mineral agar K and incubated in the presence of *n-*hexadecane vapors at 28°C. A composition of the K medium (g·L^−1^): KNO_3_ – 1.0, KH_2_PO_4_ – 1.0, K_2_HPO_4_ – 1.0, NaCl – 1.0, MgSO_4_ – 0.2, CaCl_2_ – 0.02, FeCl_3_ – 0.001, and agar – 15.0; pH 6.8–7.0 (4). A pure culture was obtained by quadrant streaking. The strain was identified initially as *Rhodococcus rhodochrous* (NCBI Taxon: 1829) on the basis of its morphological traits and enzymatic activities (5). It oxidized *n*-alkanes C_3_–C_4_ and C_11_–C_16_, thioanisole, and (–)-*trans*-carveol (data not published) and showed an extraordinary stress tolerance. It grew in the presence of 80% *n-*hexane (6); it was resistant to 5–40 mM heavy metals (data not published); and 2–5% NaCl stimulated adhesion of this strain to liquid hydrocarbons (4). The IEGM 639 strain was deposited in the Regional Specialized Collection of Alkanotrophic Microorganisms (acronym IEGM, WDCM #768, http://www.iegmcol.ru/strains/rhodoc/pyridin/r_pyridin639.html).

Cells were recovered from a lyophilized culture after storage for 1 year and grown in Luria-Bertani broth at 28°C and 160 rpm for 28 h. DNA was isolated from a pure culture using the Quick-DNA Fungal/Bacterial Microprep Kit (Zymo Research, Irvine, CA, USA), following the manufacturer’s protocol, and quantified using a Qubit 4 Fluorometer (Thermo Fisher Scientific, Waltham, MA, USA). The quality was assessed by 1% agarose gel electrophoresis. A total of 13.328 μg DNA (112 ng/μL) was obtained, and 100 ng was used for sequencing. A paired-end library (2 × 100 nucleotides [nt]) was produced using Illumina DNA Prep and sequenced on an Illumina NovaSeq 6000 instrument (Illumina, Inc., San Diego, CA, USA). The library’s quality was estimated using a fluorescence analysis and a 2100 Bioanalyzer (Agilent Technologies, Santa Clara, CA, USA). A total of 30,195,683 reads were obtained. Demultiplexing was performed using Illumina bcl2fastq (v2.20), and adapter trimming was conducted using Skewer (v0.2.2) (7). The quality of FASTQ files was analyzed with FastQC (version 0.11.5-cegat) (8). The data were assembled with SPAdes v. 3.15.4 (9). The assembly consisted of 117 contigs with a total length of 4,996,308 bp, an N50 value of 113,606 bp, a GC content of 68%, and coverage of 450×. Average nucleotide identity (ANI) and digital DNA–DNA hybridization (dDDH) were calculated using ANI Calculator (https://www.ezbiocloud.net/tools/ani) (10), Genome-to-Genome Distance Calculator 3.0 (https://ggdc.dsmz.de/ggdc.php#) (11), and Type (Strain) Genome Server (https://tygs.dsmz.de/) (12). The OrthoANIu and dDDH (*d*_4_) values were highest (98.56% and 89.2%, respectively) between the IEGM 639 and *R. pyridinivorans* DSM 44555^T^, and the IEGM 639 strain was reclassified as *R. pyridinivorans* (NCBI Taxon: 103,816). The annotation of coding sequences (CDS) was performed using PGAP 6.5 (13). Default parameters were used for all software.

A total of 4,674 CDS, 4,538 CDS with protein, 61 RNAs, and some putative genes responsible for tolerance of *R. pyridinivorans* IEGM 639 were found (Table 1).

**TABLE 1:** Putative proteins involved in resistance of *Rhodococcus pyridinivorans* IEGM 639

Type of resistance	Experimentally proved properties in details	Putative proteins	
		Product name	Quantity
Resistance to solvents and high salinity	Cells stay viable at 20 vol. % and vapors of *n*-decane (6), at 20–80 vol. % of toluene and 1-butanol (6), grow in the presence 20–80 vol. % of *n-*hexane (6), and tightly adhere to *n-*hexadecane for its oxidation in the presence of 2–5% NaCl (4)	Alkane-1 monooxygenase (EC 1.14.15.3)	2
		Cytochrome P450	12
		Flavin-dependent monooxygenase ArsO associated with arsenic resistance	1
		Aerobic carbon monoxide dehydrogenase (quinone) (EC 1.2.5.3)	9
		Alcohol dehydrogenase (EC 1.1.1.1)	7
Resistance to heavy metals	MIC^*a*^ for Cr^+6^ = 40.00 mM, MIC for Zn^2+^ = 20.00 mM, MIC for Mo^+6^ = 10.00 mM, MIC for Cd^2+^ and Pb^2+^ = 5.00 mM, and MIC for Hg^2+^ = 0.16 mM (data not published)	Cell division-associated, ATP-dependent zinc metalloprotease FtsH	1
		Cobalt/zinc/cadmium resistance protein CzcD	2
		Lead, cadmium, zinc, and mercury-transporting ATPase (EC 3.6.3.3, EC 3.6.3.5)	1
		Copper-translocating P-type ATPase (EC 3.6.3.4)	5
		Membrane-associated zinc metalloprotease	1
		YpfJ protein, zinc metalloprotease superfamily	1
		Zinc ABC transporter, ATP-binding protein ZnuC	1
		Zinc ABC transporter, permease protein ZnuB	1
		Zinc ABC transporter, substrate-binding protein ZnuA	1
		Zinc uptake regulation protein Zur	1
		Molybdopterin molybdenum transferase (EC 2.10.1.1)	3
		Periplasmic aromatic aldehyde oxidoreductase, molybdenum-binding subunit YagR	3
		Molybdenum ABC transporter, substrate-binding protein ModA	1
		Molybdenum ABC transporter permease protein ModB	1
		Molybdenum ABC transporter ATP-binding protein ModC	1
		Molybdenum cofactor guanylyltransferase (EC 2.7.7.77)/molybdopterin molybdenum transferase (EC 2.10.1.1)	1
		Lead, cadmium, zinc, and mercury-transporting ATPase (EC 3.6.3.3, EC 3.6.3.5)	5
		Mercuric ion reductase (EC 1.16.1.1)	3
		Transcriptional regulator, MerR family	7
		Lead, cadmium, zinc, and mercury-transporting ATPase (EC 3.6.3.3, EC 3.6.3.5)	5

^*a*^ MIC, minimal inhibitory concentration.

## Data Availability

This Whole-Genome Shotgun project has been deposited in DDBJ/ENA/GenBank under the accession numbers JAPWIT010000001–JAPWIT010000106. The version described in this paper is the first version available as assembly GCF_027270615.1. Raw sequence reads were deposited in the Sequence Read Archive with accession number SRR34470640 under BioSample accession number SAMN32240020 and BioProject accession number PRJNA783162.
